# Recent Advances on the Analysis of Polychrome Works of Art: SERS of Synthetic Colorants and Their Mixtures With Natural Dyes

**DOI:** 10.3389/fchem.2019.00105

**Published:** 2019-03-04

**Authors:** Anna Cesaratto, Marco Leona, Federica Pozzi

**Affiliations:** Department of Scientific Research, The Metropolitan Museum of Art, New York, NY, United States

**Keywords:** SERS, synthetic dyes, dye mixtures, sample pretreatments, nitric acid, artworks, cultural heritage

## Abstract

The development and application of proper sample pretreatments is often a key step toward the successful analysis of dyes used as artists' materials by surface-enhanced Raman spectroscopy (SERS). Complexation of the organic colorants with metal ions to dye fabrics and produce lake pigments, as well as undesired interactions with other matrix components such as substrate, binding media, fillers, and extenders, are just some of the issues that typically complicate dye identification in minute samples from invaluable artworks and museum objects. These concerns may be addressed by using, prior to SERS analysis, *ad-hoc* sample pretreatments that, in addition to increasing the technique's sensitivity, favorably affect its selectivity toward certain molecules or molecular classes. The present work describes a newly developed sample pretreatment based on the use of nitric acid that has proven crucial for the successful detection of aniline and xanthene dyes–the first synthetic organic colorants to be used in printing and painting, among other art forms–in microscopic samples from works of art such as a 19th-century silk fabric, paper cut-outs by Henri Matisse, Vincent Van Gogh's *Irises*, and Japanese woodblock prints. This treatment promotes the hydrolysis of the dye-metal bond in mordant dyes or lake pigments, resulting in a more efficient adsorption of the dye molecules on the SERS-active substrate and, hence, enabling the acquisition of high-quality spectra. In the case of synthetic colorants, this method shows advantages over hydrolysis with hydrofluoric acid–a procedure previously established for the analysis of red lakes prepared from natural dyes. The nitric acid treatment presented here may be integrated into a multi-step methodology that, by exploiting differences in solubility of various dyes and lake pigments, has enabled for the first time to successfully characterize intentional mixtures of natural and synthetic colorants of the xanthene and anthraquinone molecular classes, i.e., eosin Y and carmine, in a selection of Japanese prints of the Meiji era. The present study paves the way for the systematic identification of synthetic dyes in objects of artistic and archeological interest, even when they are present in mixtures with natural organic colorants.

## Introduction

In the last decade, surface-enhanced Raman spectroscopy (SERS) has conquered a crucial role in the detection and identification of organic colorants in works of art, especially in cases when sample size concerns prevent the use of separation techniques such as liquid chromatography (Casadio et al., 2010a, 2016; Pozzi and Leona, 2015; Pozzi et al., 2016a). As most dyes were traditionally complexed with metal ions for use in painting and textile dyeing, the application of hydrolysis pretreatments prior to SERS analysis has significantly increased the chances of successful identification of unknowns. SERS experiments on lake pigments and mordant dyes initially relied on extractions in strong acids or alkali (Leona, 2005; Chen et al., 2006; Leona et al., 2006; Whitney et al., 2006). Sulfuric and hydrochloric acids (H_2_SO_4_ and HCl) have been also used in more recent instances (Oakley et al., 2012; Mayhew et al., 2013; Roh et al., 2016) for the SERS analysis of indigo and Prussian blue, as well as yellow dyes and lake pigments in oil paint, with successful results. However, despite their high yield, such methods have been shown to often result in the disruption of the matrix, leading to the formation of degradation products that inhibit the dye-nanoparticles interaction and interfere with the analysis (Leona et al., 2006; Brosseau et al., 2009a; Bruni et al., 2010). To circumvent this issue, researchers have proposed gentler extraction procedures, and non-extractive *in-situ* hydrolysis methodologies that, while still enabling dye detection and identification in most cases, do not cause as severe a degradation of the substrate and analyte itself. These alternative methods, sometimes tailored to the sample color (Bruni et al., 2011; Pozzi et al., 2012a), typically involve removal of the dye using milder extracting reagents, such as combinations of weaker acids, organic solvents, and chelating agents (Leona et al., 2006; Brosseau et al., 2009a; Bruni et al., 2011). In some instances, researchers have followed experimental protocols in which samples are just briefly exposed to room temperature acid vapor instead of being immersed in heated solutions (Leona and Lombardi, 2007). Among the latter, a hydrolysis treatment based on the use of hydrofluoric acid (HF) has proven successful for the ultrasensitive detection of lake pigments obtained from natural dyes (Leona et al., 2006). In this procedure, briefly exposing the sample to HF vapor causes the dye-metal bond of lake pigments and mordant dyes to hydrolyze. The dye molecules are thus released into the aqueous silver colloid used as SERS-active substrate and, being water soluble, adsorb efficiently onto the nanoparticles' surface, resulting in a significant enhancement of the signal intensity without disruption of the dyes' molecular structure. Following presentation of the HF hydrolysis, its advantages and drawbacks have been systematically studied in series of samples removed from a wide variety of art objects (Pozzi et al., 2012b). In the subsequent years, this methodology has been validated as a fundamental step for the analysis of red lakes of natural origin (Pozzi et al., 2013a, 2014), although applications to the characterization of flavonoid-based yellow lakes (Cesaratto et al., 2016), weld-dyed silk fibers (Jurasekova et al., 2008; Corredor et al., 2009), and aniline-dyed textiles (Woodhead et al., 2016) have also been reported. This pretreatment was shown to be necessary even when using laser ablation (LA)-SERS, which entails an otherwise dry procedure, for the analysis of red lake-containing paint layers in cross sections (Cesaratto et al., 2014) and yellow lakes (Cesaratto et al., 2016).

As highlighted in a recent paper on the topic (Casadio et al., 2016), the application of SERS to the analysis of synthetic organic colorants in works of art has not been fully investigated yet. To fill this gap, the present work focuses specifically on aniline and xanthene dyes, which are characterized by a strong fluorescence in the visible range that often obscures the inherently weaker Raman signal. These colorants have high SERS cross sections and their SERS spectra are well-known, which makes them ideal candidates for the development and testing of new methodological approaches. Moreover, aniline and xanthene dyes are of particular interest as they were the first synthetic organic colorants to be widely used in printing and painting, among other art forms. The spectrum of crystal violet was first presented in the early developmental stages of the SERS technique (Jeanmaire and Van Duyne, 1977), and an assignment of its most distinctive bands was carried out a decade ago by means of density functional theory (DFT) (Cañamares et al., 2008). Rhodamine 6G has been used for single-molecule experiments (Kneipp et al., 1997; Nie and Emory, 1997), while rhodamine B as a tag in biosensors (Fang et al., 2008). Also, the SERS spectrum of eosin Y has been studied in depth (Narayanan et al., 1994; Whitney et al., 2006; Greeneltch et al., 2012), while phloxine has received only minor attention (Narayanan et al., 1996).

Despite the abundant work on reference synthetic dyes available in the literature, examples of SERS identification of these materials in museum objects are still rare (Brosseau et al., 2009b; Cesaratto et al., 2016; Woodhead et al., 2016). The hierarchical complexity of samples removed from artworks makes the identification of their coloring components not always straightforward. The possible aging of the materials under study and their chemical interactions with the support, binding media, and other matrix components such as fillers, extenders, and any additional pigments may complicate the analysis and subsequent data interpretation, and so does the use of colorants in their “laked” or “mordanted” form and not as free dyes. All these factors need to be accounted for when SERS analysis of natural and synthetic dyes in art-related samples is attempted. In this context, the present work aims to further expand a relatively recent study on anthraquinone lake pigments and textile dyes (Pozzi et al., 2012b) by focusing on sample pretreatments specifically tailored to the characterization of aniline (crystal/methyl violet and rosaniline/pararosaniline) and xanthene (rhodamine B, rhodamine 6G, eosin Y, and phloxine) colorants in samples from art objects. Case studies presented include the SERS analysis of aniline dyes in purple paper cut-outs by Henri Matisse and in a 19th-century silk fabric, and the characterization of xanthene dyes and lakes in a selection of pink Matisse cut-outs, in Vincent Van Gogh's *Irises*, and in Japanese woodblock prints. Our study shows that, unlike dyed paper, SERS of aniline dyes on fabrics generally requires a sample pretreatment, and that, similarly, xanthene dyes and lakes display a significant SERS enhancement when treated with a nitric acid (HNO_3_) solution. Remarkably, application of this sample pretreatment has enabled to obtain, for the first time, great results from the SERS analysis of eosin Y and phloxine in actual works of art.

Furthermore, the introduction of an additional stage in the two-step SERS procedure previously proposed by some of the authors (analysis of the sample as is and upon HF hydrolysis) (Pozzi et al., 2012b), i.e., hydrolysis with HNO_3_, has enabled the successful characterization of eosin-carmine binary mixtures in a selection of Japanese woodblock prints of the Meiji era. Interestingly, this represents the first instance in which an intentional combination of natural and synthetic dyes that do not naturally occur in mixtures has been attested in historical samples. The analytical procedure employed exploits both differences in solubility between the dyes and lake pigments present in the samples, as well as other factors such as pH and the choice of aggregating agents, enabling to selectively detect one component of the mixture over another.

While SERS has been deemed valuable for its high sensitivity and for the little sample preparation needed prior to analysis, its ability to resolve mixtures of colorants, especially if belonging to different molecular classes, has not been fully explored yet (Pozzi and Leona, 2015). This is a rather substantial problem, as plant and insect dyes are often found as complex mixtures of naturally occurring products and, even when dealing with single-component dyes such as synthetics, the possibility of intentional mixtures on the artist's or paint supplier's part cannot be ruled out (Kirby and White, 1996). Unlike chromatographic techniques such as high-performance liquid chromatography (HPLC), SERS is not able to separate the different species present in a mixture for individual detection and quantification. Moreover, surface chemistry factors may lead to the preferential adsorption of one dye over another onto the nanoparticles surface, resulting in skewed spectroscopic data. To date, various studies have examined the contribution of individual mixture components to the SERS spectra for a few selected cases of closely related dye molecules: among the reds, alizarin and purpurin (Snowden et al., 2004; Whitney et al., 2007); alizarin and lac dye (Whitney et al., 2007); alizarin and carminic acid (Murcia-Mascarós et al., 2005); various binary mixtures of the above-mentioned anthraquinone colorants and brazilwood (Pozzi et al., 2016b); and, among the yellows, luteolin and apigenin (Corredor et al., 2009; Cesaratto et al., 2016). To overcome the lack in separation capabilities, a few preliminary studies have explored the coupling of SERS with thin layer chromatography (TLC) to resolve and characterize mixtures of alizarin, purpurin, and carminic acid (Brosseau et al., 2009b), the alkaloid chromophores of the *Peganum Harmala* plant extract (Pozzi et al., 2013b), and the components of ballpoint pen inks (Geiman et al., 2009). Furthermore, TLC-SERS was applied to the study of four constituents of the first synthetic dye, mauve (Cañamares et al., 2014). More recently, an online hyphenated system combining HPLC with photodiode array (PDA) and SERS detection was proven suitable to provide a detailed electronic and vibrational characterization of natural dyes in mixtures (Zaffino et al., 2016). It is worth noting that both TLC-SERS and HPLC-SERS entail extraction of the dye components from the sample, which greatly limits the applicability of the technique to artworks and irreplaceable objects due to the relatively large amount of specimen required. However, due to the issues discussed above, successful applications of regular SERS to the detection of dye mixtures in samples from art objects are also quite infrequent. Examples in the literature include the analysis of cochineal and brazilwood mixtures in historical textile samples (Idone et al., 2013); the simultaneous identification of madder and cochineal in red lake samples from paintings by Pierre-Auguste Renoir and Édouard Manet (Pozzi et al., 2014); and the characterization of a mixture of rhodamine B and rhodamine 6G in a pink pastel from Mary Cassatt's pastel box (Brosseau et al., 2009b). Yet, to date, the simultaneous SERS detection and identification of non-closely related dyes in samples from artworks has never been accomplished. Therefore, the results reported here represent a great advancement in the field, and may serve as a reference when the SERS technique is used for the study of synthetic dyes in modern and contemporary art objects.

## Materials and Methods

### Reference Materials and Artworks

Rosaniline was obtained from Eimer and Amend. Pararosaniline, crystal violet, rhodamine 6G, and eosin Y were purchased from Sigma Aldrich. Methyl violet was obtained from Fluka Chemical Corp. Rhodamine B was purchased from Fischer Scientific. Phloxine was obtained from Acros Organics. Microscopic samples of roughly equivalent size were removed from several works of art, as described in Table 1. Samples studied include: paper fibers from a collection of gouache paper samples from Henry Matisse's cut-out paintings (The Museum of Modern Art, New York); paper fibers from a polychrome print, *Ladies Sewing* (*Kijo saiho no zu*), by Adachi (Shosai) Ginko (1887, The Metropolitan Museum of Art, New York, accession number JP3272); paper fibers from a collection of ten late Edo-early Meiji woodblock Japanese prints (1860-1898, private collection); paint samples from Vincent Van Gogh's *Irises* (1890, The Metropolitan Museum of Art, New York, accession number 58.187).

**Table 1:** List of samples analyzed by SERS in the present study.

**Samples**	**Art objects**	**Artistic techniques**	**SERS characterization**
Violet 1 bis	Henri Matisse's cut-outs	Gouache on paper	Faded crystal/methyl violet
Violet 4	Henri Matisse's cut-outs	Gouache on paper	Highly faded crystal/methyl violet and/or rosaniline/pararosaniline
Purple silk	Nineteenth-century silk fabric	Dyed silk	Faded crystal/methyl violet
M1	Henri Matisse's cut-outs	Gouache on paper	Rhodamine B
M4	Henri Matisse's cut-outs	Gouache on paper	Rhodamine B and 6G
R5	Henri Matisse's cut-outs	Gouache on paper	Eosin Y
P/R3	Henri Matisse's cut-outs	Gouache on paper	Phloxine
VG1	Vincent Van Gogh's *Irises*	Oil painting	Eosin Y
Various	Japanese woodblock prints	Polychrome prints	Eosin Y and carmine

*The sample names, art objects from which the samples were removed, artistic techniques, and dyes identified are indicated*.

### Silver Colloid Preparation

A monodisperse colloidal suspension of silver nanoparticles was synthesized by microwave-supported glucose reduction of silver sulfate in the presence of sodium citrate as a capping agent. Details on the synthetic procedure and physicochemical properties of this type of colloids (Leona, 2009), as well as a comparison with other silver substrates used for SERS in the cultural heritage field (Pozzi and Leona, 2015; Pozzi et al., 2016a), are reported elsewhere in the literature. Silver nanoparticles produced by this microwave-assisted methodology have λ_max_ = 401 nm, FWHM = 50 nm, and a narrow nanoparticle size range of 3–10 nm, leading to great efficiency, high stability over time, and reproducible SERS results. A 5x-concentrated colloid, obtained by centrifuging 1 mL of nanoparticle suspension and replacing 950 μL of the supernatant with 150 μL of 18 MΩ water, was used for all analyses reported in this work.

### Sample Pretreatments and SERS Procedure

**Figure 1:**
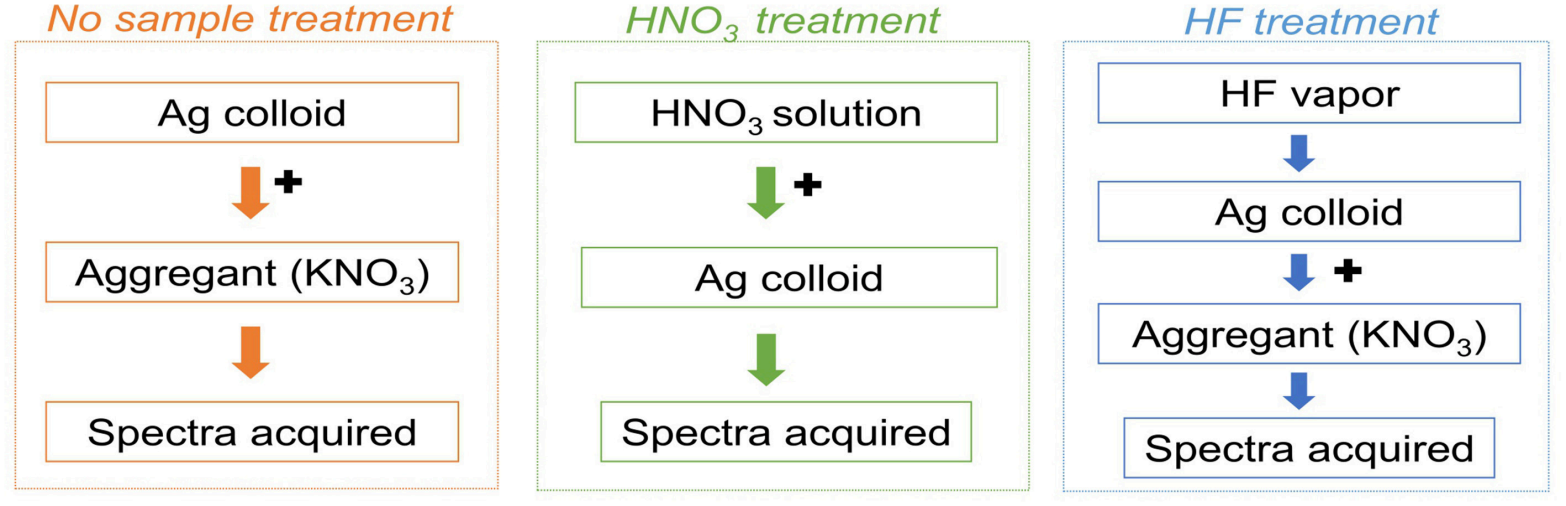
Sample treatments used for the SERS analysis reported in this study.

Three different SERS procedures have been used, as schematically illustrated in Figure 1. These three methodologies, all carried out at room temperature, may be combined into a multi-step approach and applied in sequence to the same sample. In the first step, the sample is placed on a polyethylene holder and analyzed as is, after being covered with a 2 μL drop of colloid and 0.4 μL of 0.5 M KNO_3_ to induce nanoparticle aggregation. Following acquisition of a SERS spectrum, the colloid droplet is removed with a micropipette or let evaporate. In the second step, the sample is treated with 1 μL of 1% HNO_3_ aqueous solution, and a 2 μL drop of colloid is then dropcasted onto the sample. Upon acquisition of a SERS spectrum, the colloid droplet is again removed or let evaporate. In the third and last step, the sample is exposed to HF vapor for 5 min in a closed microvial (Leona et al., 2006), and a SERS spectrum is acquired upon addition of colloid and 0.5 M KNO_3_. In the latter step, the HF-containing microreactor is prepared by placing as little as 10 μL of acid at the bottom every 4–5 days, greatly reducing the risks associated with the use of this reagent. Moreover, it is worth highlighting that, besides promoting the hydrolysis of the dye-metal bond in mordant dyes and lake pigments, the HNO_3_ simultaneously acts as an aggregant for the silver nanoparticles, thus enabling to combine into a single step what, in most cases, has been accomplished in two separate phases of the analytical protocol through the use of different chemicals. In addition to a longstanding and well-documented use of nitrate ions in SERS to activate silver colloids by inducing nanoparticle aggregation, the selection of HNO_3_ finds reason in the fact that, as previously discussed, other strong acids such as HCl have been reported to cause an excessive disruption of the host material or competition for the adsorption onto the metal surface (Leona et al., 2006; Bruni et al., 2010), ultimately preventing dye detection and identification.

### Instrumentation

A Bruker Senterra Raman instrument equipped with a charge-coupled device (CCD) detector was used to acquire the SERS spectra, exciting the specimens at 488 and 633 nm by means of solid state laser and He-Ne lasers, respectively. The laser beam was focused through a 20x long working distance microscope objective just below the surface of the silver nanoparticle droplet. The laser power at the sample was kept below 0.2 mW (λ_exc_ = 488 nm) and 1 mW (λ_exc_ = 633 nm) to avoid thermal damage. The spectra reported in this article were acquired with a resolution of about 3–5 cm^−1^, integrating the signal two times for 15 s each.

## Results and Discussion

All the dyes examined in this work, with the exception of rhodamines, have been characterized by means of a solid state laser providing excitation at 488 nm. This wavelength is typically the most suitable to produce high-quality SERS spectra, as it matches both the silver colloids' plasmon resonance and most red dyes' optical absorption, leading to a significant increase in the technique's sensitivity (Leona, 2009). Among the synthetic colorants identified in the present study, only rhodamines were found to yield better SERS results when excited at 633 nm using a He-Ne laser, likely because, in this case, the resonance between the laser excitation and the dyes' optical absorption–shifted toward higher wavelengths, i.e., around 500–550 nm–plays a more crucial role toward a successful analysis compared to other factors, including resonance with the nanoparticles' absorption maximum. Unless otherwise specified, all the spectra in this article are shown without modifying their relative intensities, in order to enable an easy and effective comparison of the results obtained using the three methodologies described above (sample not treated, sample treated with HNO_3_ solution, and sample treated with HF vapor). Illegible spectra that displayed very low relative enhancements have been multiplied by a certain factor to help in inspection; such factor is reported in the corresponding graphs for clarity. Three spectra were typically collected from each sample to ensure reproducibility of the results in terms of band position. In each graph, the reference spectra of commercial pure dyes and lake pigments, appearing as dashed lines, are reported along with those collected from the art samples investigated to provide evidence of the colorants identified. Wavenumbers of the main peaks are marked in the figures to facilitate visual examination. Usually, spectra acquired without any pretreatment and after hydrolysis with HNO_3_ and HF are enclosed in the same graph, to make the effects of the various methodologies easier to compare.

### Aniline Dyes

#### Crystal/Methyl Violet and Rosaniline/Pararosaniline

**Figure 2:**
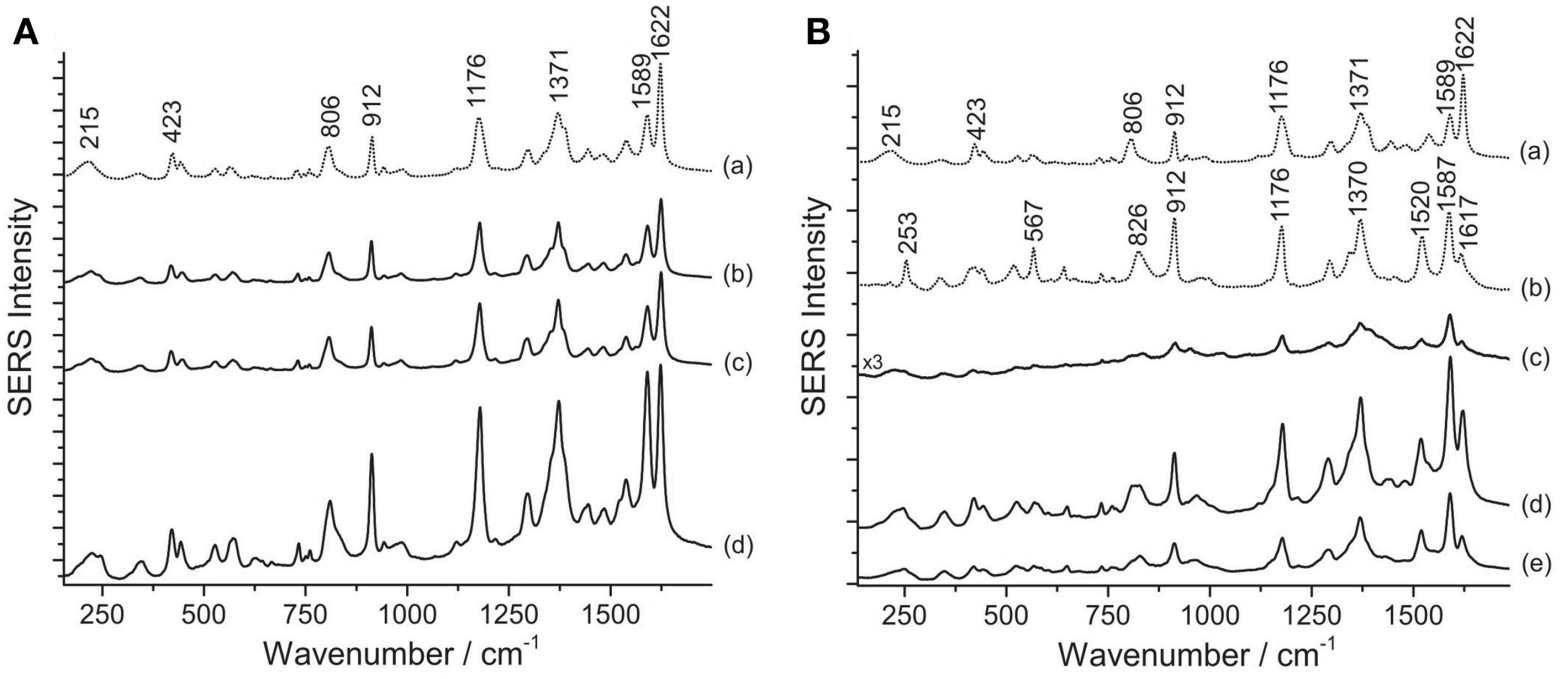
SERS spectra of aniline dyes from some of Matisse's paper cut-outs (λ_exc_ = 488 nm). **(A)** Spectrum of reference crystal/methyl violet (a) compared with spectra acquired from sample Violet 1 bis without any treatment (b), after HNO_3_ treatment (c), and after HF treatment (d). **(B)** Spectra of reference crystal/methyl violet (a) and pararosaniline (b) compared with spectra acquired from sample Violet 4 without any treatment (c), after HNO_3_ treatment (d), and after HF treatment (e). Signals of faded crystal/methyl violet are detected at 215, 423, 806, 912, 1176, 1371, 1589, and 1622 cm^−1^, while signals of highly faded crystal/methyl violet or pararosaniline are detected at 215, 253, 423, 567, 806, 826, 912, 1176, 1370, 1520, 1589, and 1622 cm^1^.

SERS investigation of some of the samples removed from Henry Matisse's purple cut-outs show the presence of aniline dyes. It is worth noting that the SERS spectra of crystal/methyl violet and rosaniline/pararosaniline are respectively identical (Geiman et al., 2009; Cesaratto et al., 2017). A definitive assignment of the symmetry and normal modes of the spectrum of crystal violet can be found in the literature (Cañamares et al., 2008). As explained in a recent article, SERS spectra of crystal/methyl violet are highly dependent on the level of photo-degradation of the material under study (Cesaratto et al., 2017). In particular, the SERS spectrum of crystal violet assumes some of the characteristic features of pararosaniline as a result of the progressive N-demethylation induced by light exposure. Accordingly, Matisse's cut-out sample Violet 1 bis shows the typical signals of faded crystal/methyl violet–materials that can be easily detected even without sample treatments (Figure 2A); in this case, in fact, application of the HNO_3_ hydrolysis did not affect the SERS signal intensity. On the other hand, the HF treatment slightly enhanced the signal but, at the same time, it also altered the relative ratio of peaks at 1589 and 1622 cm^−1^. As such ratio is indicative of the material's photo-induced fading level (Cesaratto et al., 2017), this factor must be taken into consideration when selecting the most suitable treatment for samples that are likely to contain aniline dyes.

Compared with the latter, Matisse's cut-out sample Violet 4, identified as highly faded crystal/methyl violet or rosaniline/pararosaniline, displays a different behavior (Figure 2B). In this case, the hydrolysis with HNO_3_ was found to improve the otherwise weak SERS signal. The higher fading levels exhibited by the dyes contained in this sample may be responsible for the lower spectral quality observed when the specimen is not treated prior to SERS analysis. Interestingly, the ratio of peaks at 1589 and 1622 cm^−1^ appeared to remain overall stable regardless of the sample treatments applied. Based on these data, a hypothesis may be put forward that aniline colorants on paper may have been used here as free dyes; therefore, successful characterization can be accomplished even without sample pretreatments, while HNO_3_ might help enhance the signal in case of highly degraded samples, as also observed in a study of Japanese woodblock prints recently published (Cesaratto et al., 2017).

**Figure 3:**
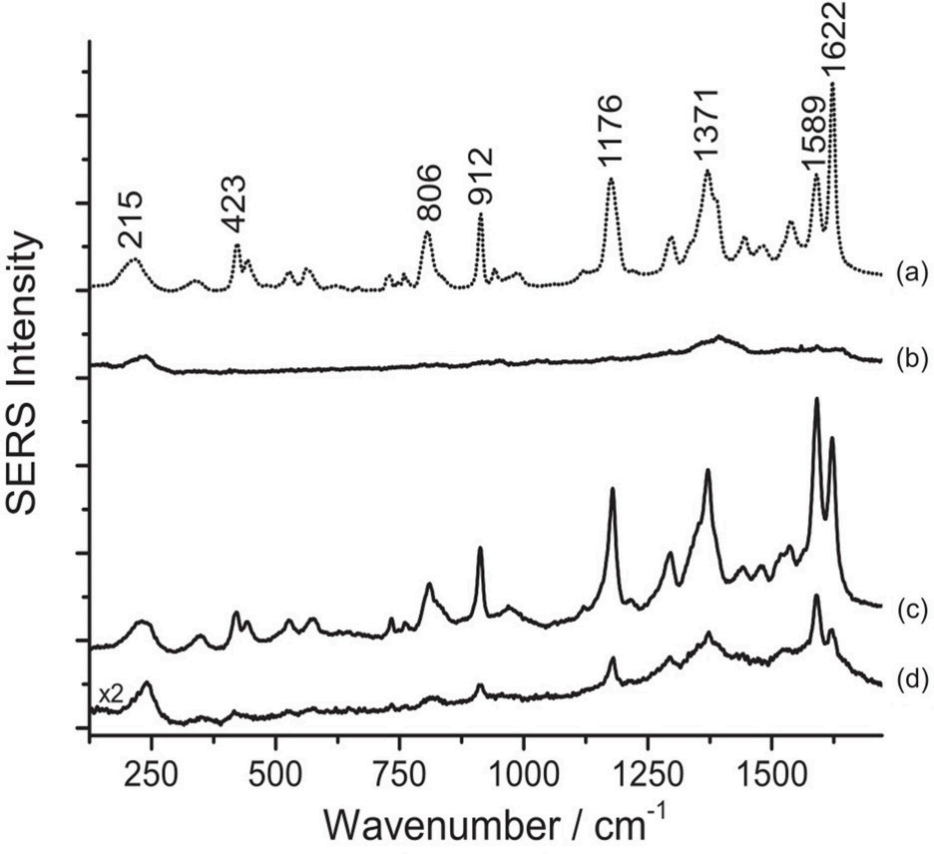
SERS spectra of a nineteenth-century purple silk fabric (λ_exc_ = 488 nm). Spectrum of reference crystal/methyl violet (a) compared with spectra acquired from the dyed silk sample without any treatment (b), after HNO_3_ treatment (c), and after HF treatment (d). Signals of faded crystal/methyl violet are detected at 215, 423, 806, 912, 1176, 1371, 1589, and 1622 cm^−1^.

Contrarily to what was observed on the Matisse cut-out samples, SERS analysis of crystal/methyl violet in a 19th-century silk fabric was only possible upon treatment with acids. As shown in Figure 3, when the sample is treated with HNO_3_, the signal-to-noise ratio drastically increases, making even the small features that are characteristic of these dyes clearly detectable. Our observations regarding the necessity of a sample pretreatment for aniline-dyed textiles are in accordance with experimental results presented in a recent paper by Woodhead et al. (2016). Even if both the HF and HNO_3_ treatments enabled dye identification by hydrolyzing the dye-mordant complex, the latter method was found to be more effective and less time consuming than the HF hydrolysis. Indeed, as already suggested by one of the authors (Pozzi et al., 2012b), the HF might cause the silk proteins to be released into solution and interfere with dye adsorption onto the silver nanoparticles, resulting in a significant signal broadening and deterioration of the SERS spectra.

### Xanthene Dyes and Lakes

#### Rhodamine

**Figure 4:**
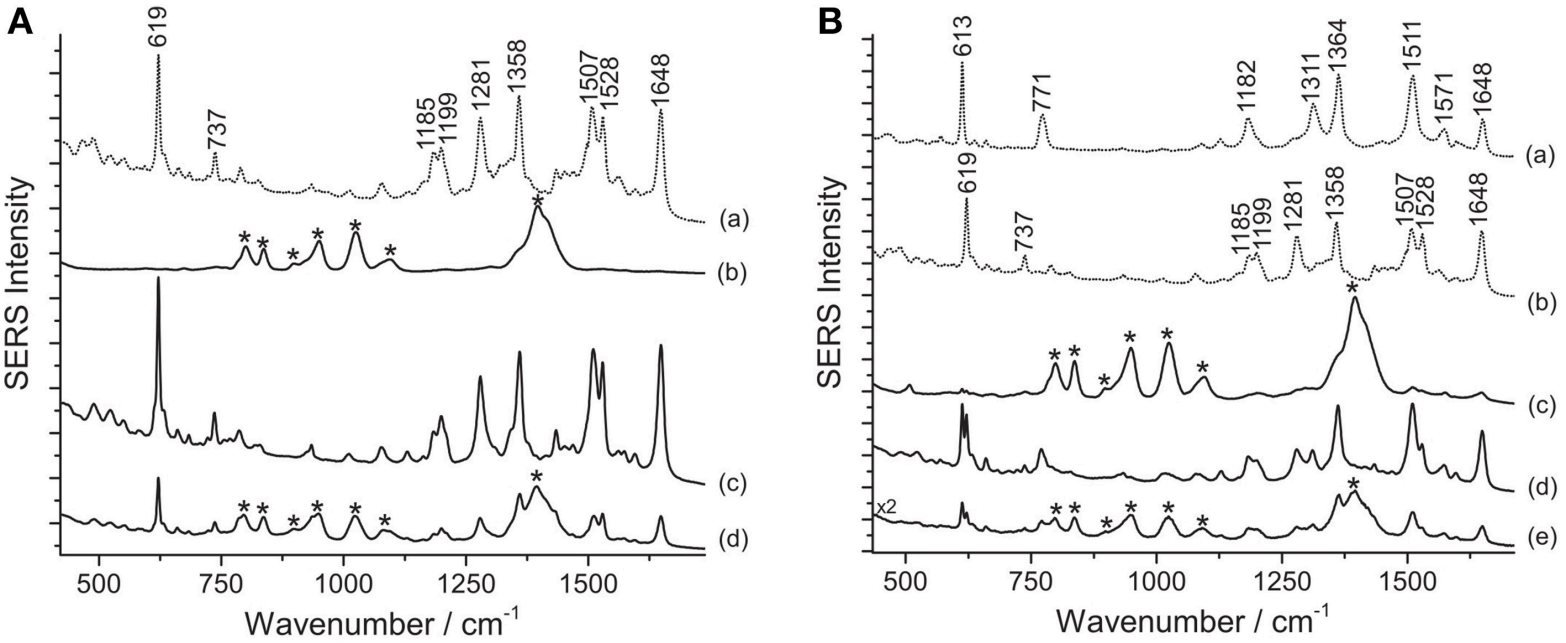
SERS spectra of xanthene dyes from some of Matisse's paper cut-outs (λ_exc_ = 633 nm). **(A)** Spectrum of reference rhodamine B (a) compared with spectra acquired from sample M1 without any treatment (b), after HNO_3_ treatment (c), and after HF treatment (d). **(B)** Spectra of reference rhodamine 6G (a) and rhodamine B (b) compared with spectra acquired from sample M4 without any treatment (c), after HNO_3_ treatment (d), and after HF treatment (e). Signals of rhodamine B are detected at 619, 737, 1185, 1199, 1281, 1358, 1507, 1528, and 1648 cm^−1^, while signals of a mixture of rhodamine B and rhodamine 6G are detected at 613, 619, 737, 771, 1182, 1199, 1281, 1311, 1362 (combination band), 1509 (combination band), 1528, and 1648 cm^−1^. Spurious bands due to citrate are marked with an asterisk.

Various dyes belonging to the xanthene group are responsible for the bright pink color shades of a second selection of paper cut-outs by Henri Matisse examined in this work. An assignment of the observed peaks for rhodamine 6G and B is reported in the literature (Jensen and Schatz, 2006; Lin et al., 2015). SERS spectra of rhodamine-containing samples, i.e., M1 and M4 (Figure 4), were acquired by means of a 633 nm laser. This excitation wavelength quenches the fluorescence background arising from the unadsorbed dye molecules more effectively than other shorter laser emissions and, due to resonance effects, greatly enhances rhodamine B's and 6G's characteristic peaks at 619 and 613 cm^−1^, respectively, making conclusive identification easier. This is especially useful when the two dyes are present in a mixture, as in sample M4 (Figure 4B). As shown in Figure 4, the application of the HNO_3_ treatment was crucial to produce a clean spectrum of rhodamine B from sample M1. Without treatment, this sample does not show any characteristic bands ascribable to the colorant, while the spurious signals of citrate ions from the silver colloid are present along with those of the dye upon HF hydrolysis. Similarly, the rhodamine B and rhodamine 6G mixture in M4 requires a sample pretreatment with HNO_3_ to enhance the dyes signal over the citrate bands. It is worth noting that, in the latter case, the sample gives rise to a SERS spectrum, albeit weak and dominated by citrate bands, even when it is not pretreated. In the spectrum shown in Figure 4B, the band at 1362 cm^−1^ is a combination of two signals at 1364 cm^−1^ (rhodamine B) and 1358 cm^−1^ (rhodamine 6G), while the band at 1509 cm^−1^ is a combination of peaks at 1511 cm^−1^ (rhodamine B) and 1507 cm^−1^ (rhodamine 6G), respectively. In the past, the use of mixtures of rhodamine B and 6G as artists' materials has already been confirmed by SERS in a pink pastel from a pastel box belonging to Mary Cassatt from the Boston Museum of Fine Arts (Brosseau et al., 2009b). The spectra shown in the above-mentioned article are characterized by strong peaks attributed to citrate, consistently with the fact that the sample had not been treated prior to SERS analysis.

According to the results obtained from analysis with X-ray fluorescence (XRF) spectroscopy, rhodamine B in sample M1 is present as a phosphorus (P)–molybdenum (Mo)–tungsten (W) lake, as it is common in commercial formulations of the pigment. The acidic conditions reached when using HNO_3_ appear to be ideal to induce the hydrolysis of the rhodamine lake, making the free colorant available for adsorption onto the silver nanoparticles. Based on XRF data, however, it is not possible to determine whether rhodamine was used in sample M4 in the form of a lake pigment, while the SERS results obtained after the first analytical step (no hydrolysis) seem to indicate that at least a fraction of the dye molecules are solubilized in the colloidal droplet and able to adsorb onto the SERS substrate. However, the detection of citrate peaks in spectra collected without hydrolysis indicates that the adsorption process was inefficient and/or incomplete. For both samples, the HNO_3_ treatment induces the release of the free dye molecules and promotes most efficiently their adsorption on the SERS-active surface, resulting in the complete replacement of the citrate bands previously observed in the spectra with the dye signals. On the other hand, the results obtained for M1 and M4 upon HF hydrolysis seem to point toward an incomplete substitution of the citrate ions with the dye molecules on the nanoparticles surface.

#### Eosin Y and Phloxine

**Figure 5:**
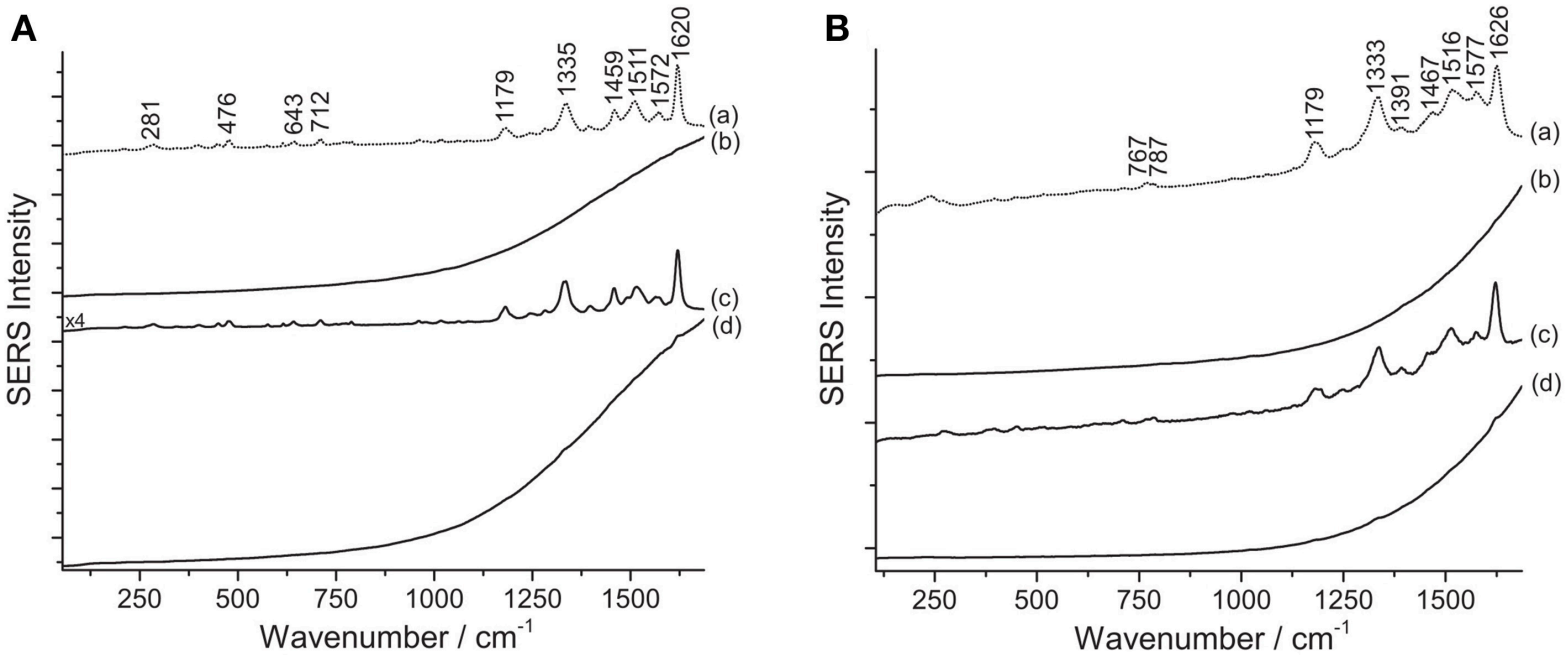
SERS spectra of xanthene dyes from some of Matisse's paper cut-outs (λ_exc_ = 488 nm). **(A)** Spectrum of reference eosin Y (a) compared with spectra acquired from sample R5 without any treatment (b), after HNO_3_ treatment (c), and after HF treatment (d). **(B)** Spectrum of reference phloxine (a) compared with spectra acquired from sample P/R3 without any treatment (b), after HNO_3_ treatment (c), and after HF treatment (d). Signals of eosin Y are detected at 281, 476, 643, 712, 1179, 1335, 1459, 1511, 1572, and 1620 cm^−1^, while signals of phloxine are detected at 767, 787, 1179, 1333, 1391, 1467, 1516, 1577, and 1626 cm^−1^.

Results from SERS analysis suggest that Matisse extensively used eosin Y and phloxine to produce a bright pink color shade often observed in some of his cut-out paintings. An assignment of the normal modes of the spectrum of eosin Y can be found in the literature (Greeneltch et al., 2012). These two closely related dyes display a similar response toward the different sample treatments. Indeed, in both cases, the use of HNO_3_ proved essential to achieve dye identification, as can be clearly observed for sample R5, containing eosin Y, and sample P/R3, consisting of phloxine (Figure 5). When samples are analyzed as is, a strong fluorescence emission arises, which might be ascribable to the unsuccessful adsorption of the dye on the SERS substrate. Similarly, the HF-treated samples give rise to a highly fluorescent background, in which even the main peaks at ~1330 and ~1620 cm^−1^ are barely visible.

**Figure 6:**
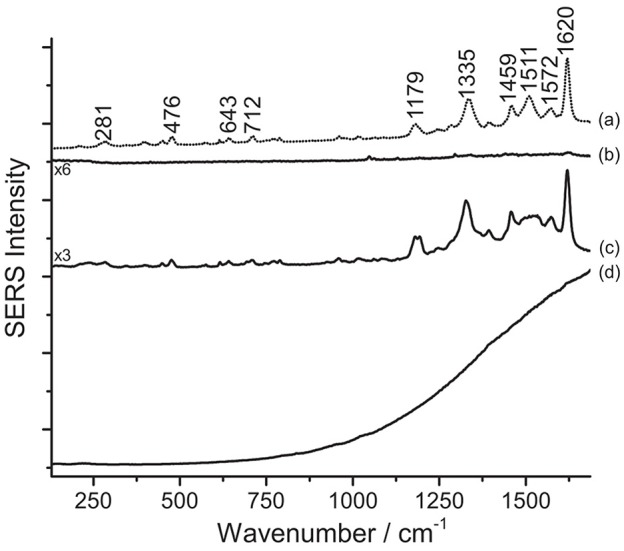
SERS spectra of an eosin lake from Van Gogh's *Irises* (λ_exc_ = 488 nm). Spectrum of reference eosin (a) compared with spectra acquired from the Van Gogh paint sample without any treatment (b), after HNO_3_ treatment (c), and after HF treatment (d). Signals of eosin Y are detected at 281, 476, 643, 712, 1179, 1335, 1459, 1511, 1572, and 1620 cm^−1^.

As previously reported in the literature, in the present work, too, SERS analysis played a key role in shedding light on the original appearance of works of art that have undergone severe fading overtime. SERS of a sample removed from Van Gogh's *Irises*, in The Met's holdings, revealed the presence of eosin Y, suggesting that the currently white painting background may have been, in the artist's original intention, of a bright pink hue. Similarly to some of the samples discussed above, the dye, in this case, could be only identified upon HNO_3_ treatment, as shown in Figure 6.

One key difference may be noticed in spectra of eosin-containing untreated samples reported in Figures 5, 6: while Matisse's cut-outs show a strong fluorescent background, the eosin lake found in Van Gogh's sample gives rise to a weak SERS signal and poor signal-to-noise ratio. In the latter spectrum, however, a weak peak at 1620 cm^−1^ can be actually detected, which might be related to the few free dye molecules present in the sample as residues from the laking process. The observation of a high fluorescence emission in Matisse's cut-out samples and absence of it in the Van Gogh paint specimen might suggest that eosin Y and phloxine are present as free dyes in the first (for which XRF could not determine if metal ions were used as mordants) or that, at least, a portion of the colorant was not complexed. It may be hypothesized that the dye molecules may have entered the aqueous solution of the colloidal droplet, but did not properly adsorb on the nanoparticles; as an alternative, the dyes may have reacted with the matrix in which they are embedded, making them unavailable for solubilization and adsorption onto the SERS-active substrate. On the other hand, the great results obtained upon treatment with HNO_3_ are likely achieved thanks to the acidic environment provided by the acid that favors the dye adsorption on the metal surface. As regards the Van Gogh sample, it is well-known that eosin Y has been used as a lead (Pb) lake pigment by the artist, as indicated by normal Raman and scanning electron microscopy/energy-dispersive X-ray (SEM/EDX) spectroscopy (Centeno et al., 2017). In the first step of the analysis (no treatment), it was found that, when in the form of a water-insoluble lake, eosin Y cannot enter the aqueous solution of the colloidal droplet due to its scarce solubility in the medium. In the second step (hydrolysis with HNO_3_), an intense SERS signal is collected, indicating that the dye-metal bond has been completely hydrolyzed by the acid, leading to the preferential adsorption of the free dye molecules onto the silver nanoparticles. In the third step (hydrolysis with HF), the HF is able to hydrolyze the dye-metal complex in the lake pigment, although the overall chemical conditions do not enable a complete adsorption of the dye molecules on the SERS-active substrate. This results in a SERS spectrum in which the main signals of eosin Y are barely visible over a strong fluorescent background that is due to the presence, in solution, of some free dye molecules that were not adsorbed on the silver surface. To the best of the authors' knowledge, the results reported above represent the first instances in which eosin Y and phloxine are identified in actual art objects by SERS. In the past, FT-Raman spectroscopy has been used for the characterization of phloxine in José Gaudalupe Posada prints (Casadio et al., 2010b). The SERS identification of eosin Y in the Van Gogh sample opens up new frontiers for the study of eosin-containing faded paintings for which only minimally invasive sampling is allowed (Burnstock et al., 2005).

### Xanthene-Anthraquinone Mixtures

#### Eosin Y and Carmine

**Figure 7:**
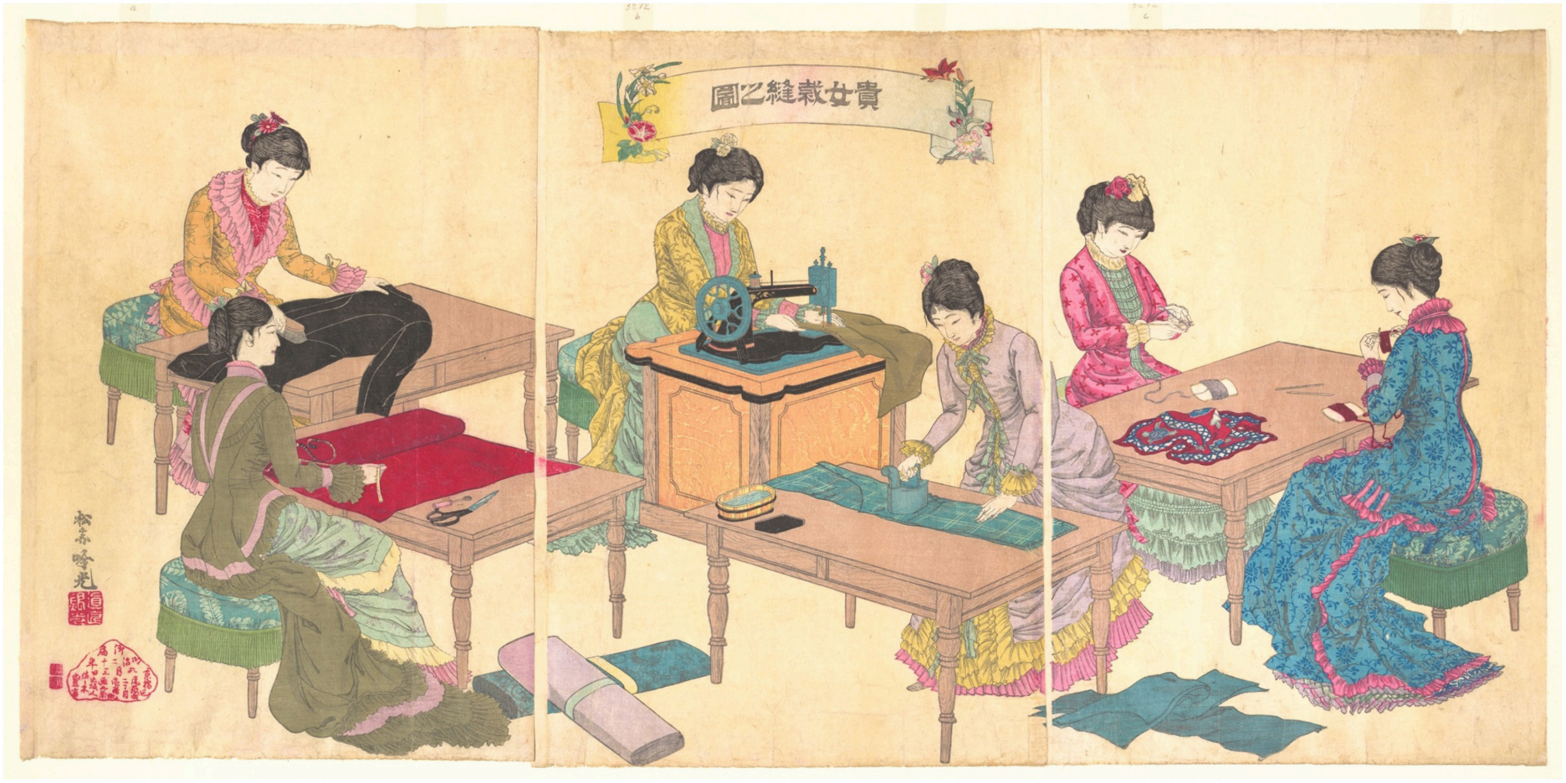
Adachi (Shosai) Ginko, *Ladies Sewing (Kijo saiho no zu)*, dated to September 3rd of 1887. Reproduced with permission of the Metropolitan Museum of Art, New York, accession number JP3272.

**Figure 8:**
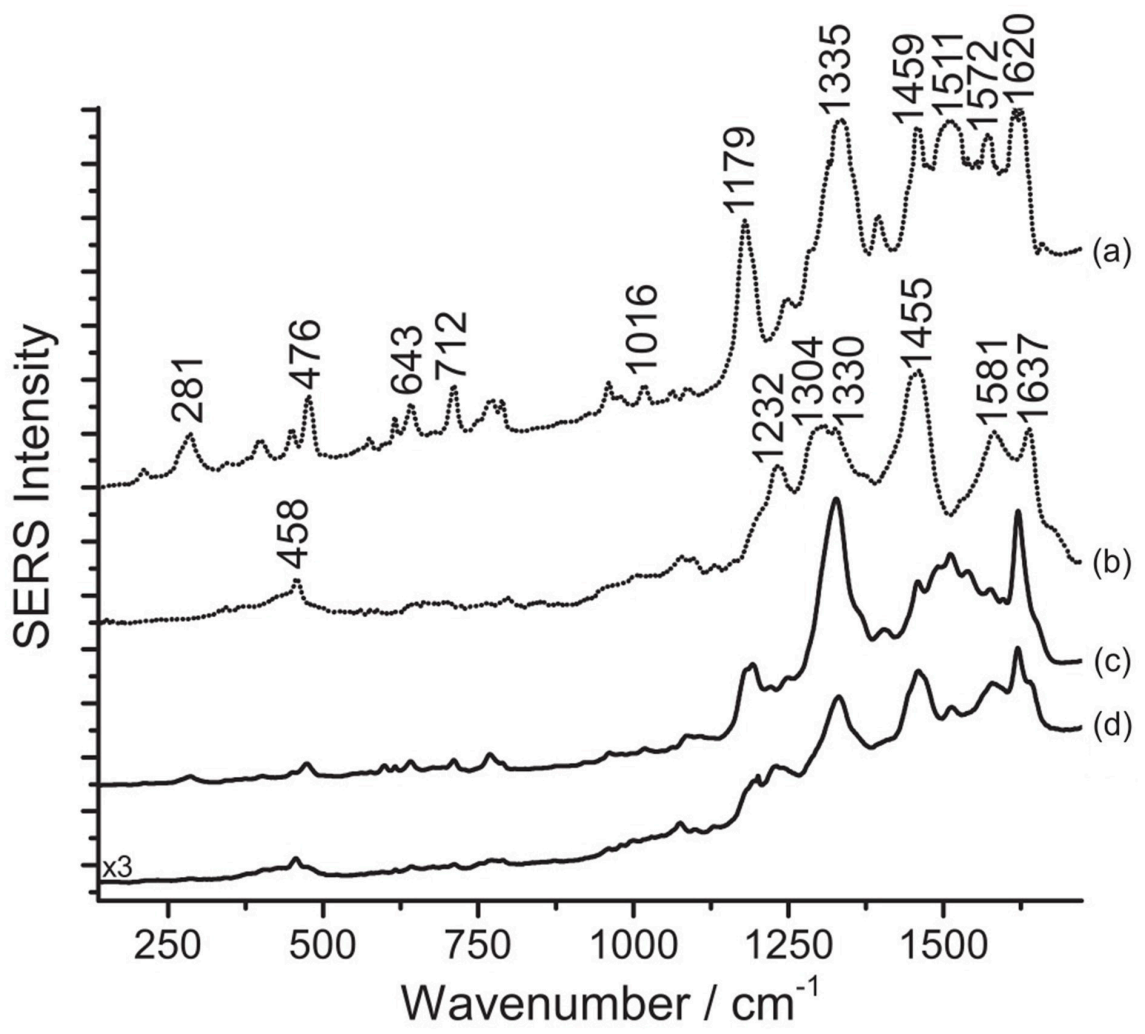
SERS spectra of a mixture of eosin Y and carmine in Japanese woodblock prints (λ_exc_ = 488 nm). Spectrum of reference eosin Y (a) and carmine lake (b) compared with spectra acquired from the print sample after HNO_3_ treatment (c), and after HF treatment (d). In spectrum (c), signals of eosin Y are detected at 281, 476, 643, 712, 1016, 1179, 1335, 1459, 1511, 1572, and 1620 cm^−1^. In spectrum (d), signals of carmine lake are detected at 458, 1232, 1330 (combination band), 1455, 1581, and 1637 cm^−1^, while some signals of eosin Y are also detected at 281, 476, 643, 712, 1179, 1332 (combination band), 1511, and 1620 cm^−1^.

As a final step of the present study, samples removed from red areas of ten Japanese prints of the Meiji era (1877-1898) were analyzed with SERS following the three-step procedure presented in this work. One of the prints examined is shown in Figure 7. It was noticed at first that, without acidic pretreatment, samples from such prints yield poor SERS data, characterized by a highly fluorescent background, and no specific peaks. On the other hand, hydrolysis with HNO_3_ leads to the preferential adsorption of eosin Y over carmine on the silver nanoparticles, as shown in Figure 8. The SERS spectra collected correlate well with previously published data of eosin Y (Narayanan et al., 1994; Whitney et al., 2006; Greeneltch et al., 2012). In particular, based on the classification reported by Whitney et al. (2006), eosin Y could be present in the samples from the Japanese prints as a free acid; in our spectra, only the peak at 1016 cm^−1^ is ascribable to the disodium salt, even if it is not one of the most characteristic for this specific form. In principle, the presence of eosin Y in its free acid form is in accordance with the fact that normal Raman spectra acquired on the same areas as those sampled for SERS are characterized by an intense fluorescence signal arising from the dye (Whitney et al., 2006). Nevertheless, the fluorescent background could also originate from the other materials present in the sample, such as additional dyes and pigments, starch, and/or the paper itself. Interestingly, after HF hydrolysis, the typical signals of carmine are also detected in the SERS spectrum (Figure 8). An assignment of the normal modes for carminic acid, the main coloring molecule of cochineal and carmine lakes, may be found in the literature (Cañamares et al., 2006).

The pH reached by treating the samples with a 1% HNO_3_ aqueous solution is evidently not sufficient to break the chemical bond between carminic acid and its substrate in the lake used by the printer, while eosin Y, more soluble in the medium, can be more easily detected. On the other hand, as the HF treatment was originally designed to hydrolyze anthraquinone-based lake pigments (Pozzi et al., 2012b), the corresponding SERS spectrum shows a strong signal for carminic acid that, as expected, is predominant compared to that of eosin Y. In the spectrum obtained upon HF hydrolysis, reported in Figure 8, the signals of both carminic acid and eosin Y can be identified. Compared to spectra acquired after hydrolysis with HNO_3_, the signals of eosin Y decrease visibly following HF treatment, as can be noted comparing spectra (c) and (d). In the absence of other studies on Japanese printmakers' practices, it is not clear if, in these cases, eosin Y and carminic acid were used as free dyes or as lakes. Experimental evidence gathered from the prints, in particular an intense signal for aluminum detected by SEM/EDX for cochineal-colored samples, suggests that cochineal might have been used as an aluminum lake. Results from SERS analysis following the three-step procedure described here confirmed that cochineal is indeed found as a lake pigment (Leona, 2009; Pozzi et al., 2012b); indeed, free carminic acid is rather soluble in water and, if present, would adsorb rapidly onto the silver nanoparticles in the first step, producing a legible SERS response, while in the present case analysis of the untreated samples gave no results. As for eosin Y, lead, typically used to produce eosin lakes in Europe (Eastaugh et al., 2004), was rarely detected. Based on the absence of lead and other elements that may have been used as laking substrates in the XRF data, it is possible to hypothesize that, in our case, eosin Y was used as a free dye by the Japanese printmaker. Accordingly to what was previously stated, the high fluorescence detected when the samples are not treated seems to indicate the presence of eosin Y as a free dye in the material examined. However, the use of a mixture of the dye along with the corresponding lake pigment cannot be excluded. In both cases, the HNO_3_ treatment enables an efficient interaction between the silver nanoparticles and the dye molecules, resulting in the best and most consistent analytical results.

As a final remark, the experiments performed showed that different results are obtained when the step order, in particular steps 2 and 3, is inverted. Indeed, to accomplish detection of eosin successfully, it is crucial to carry out the HNO_3_ hydrolysis before treatment with HF. When the latter is used first, the carmine lake is hydrolyzed and free carminic acid is brought into solution. Likely due to the two molecules' differences in terms of solubility and affinity for the metal substrate, carminic acid then competes with eosin for adsorption onto the silver nanoparticles, resulting in the anthraquinone signals becoming predominant in the SERS spectrum.

## Conclusions

In the present study, three SERS approaches for the selective detection and conclusive identification of synthetic dyes in samples from artworks were evaluated and compared, i.e., SERS on silver nanoparticles without any preliminary sample treatment, upon hydrolysis with HNO_3_, and after treatment with HF. These three methodologies may be conveniently incorporated into a three-step analytical procedure, in which they can be performed in sequence on the same microscopic sample upon physical removal or evaporation of the colloidal droplet after each step.

The synthetic dyes examined in this work, belonging to the aniline and xanthene molecular classes, show a high fluorescence emission in the visible range of the electromagnetic spectrum, which often obscures the intrinsically weaker Raman signal, and are therefore ideal candidates for SERS. Being good SERS scatterers, these colorants have been used in previous studies to test innovative methodological approaches of interest to the biology and forensic science fields. In addition, aniline and xanthene dyes are greatly relevant to cultural heritage research, as it is attested by the fact that they were the first synthetic organic colorants to be widely used in printing and painting, among other art forms. Their extensive occurrence as artists' materials and the lack of reliable, minimally invasive identification methods to date prompted the authors to design a SERS procedure specifically tailored to their detection and characterization in samples removed from works of art and objects of archaeological and historical significance.

The present study shows that aniline dyes on paper give rise to good quality SERS spectra even without pretreating the sample, although hydrolysis with HNO_3_ may help improve the signal-to-noise ratio in spectra from highly degraded specimens. In the case of silk dyed with crystal/methyl violet, treatment of the sample with acids was crucial to induce the hydrolysis of the dye-mordant complex; HNO_3_ was found to be more effective than HF for this purpose.

When dealing with xanthene dyes and lakes, the application of a HNO_3_ pretreatment step is highly recommended. When xanthenes are present as free dyes, the use of HNO_3_ enables the efficient adsorption of the dye molecules onto the SERS-active substrate, resulting in a significant enhancement of the SERS signal combined with the complete suppression of colloid-related citrate peaks (for rhodamine) and background fluorescence (for eosin Y and phloxine). In the case of xanthene lakes, the main purpose of using the HNO_3_ is to hydrolyze the dye-metal complex in the lake pigment and release the free dye molecules for adsorption onto the nanoparticles surface.

Interestingly, it was found that pretreating the sample with two different acids (HNO_3_ and HF) in sequence is key to the selective detection and identification of non-closely related dyes in mixtures. In this context, the present study reports, for the first time, an instance in which a multi-step SERS approach was applied to the characterization of mixtures of colorants belonging to the xanthene and anthraquinone molecular classes, i.e., eosin Y and carmine, in actual artworks. The occurrence of natural and synthetic dyes in combination cannot be ruled out when examining works by modern and contemporary artists whose studio practice is still unknown or under investigation. In these cases, the introduction of an additional step based on the use of HNO_3_ in the SERS procedure, which only requires a few additional minutes compared to the two-step methodology presented by some of the authors in an earlier article, was found to be essential to reveal such mixtures.

## Data Availability

The datasets generated for this study are available on request to the corresponding author.

## Author Contributions

AC carried out most of the experimental work and drafted the paper. FP helped with the experimental work and actively contributed to the preparation and submission of the manuscript. ML offered guidance and supervision throughout the project.

### Conflict of Interest Statement

The authors declare that the research was conducted in the absence of any commercial or financial relationships that could be construed as a potential conflict of interest.

## References

[B1] Brosseau C. L. Gambardella A. Casadio F. Grzywacz C. M. Wouters J. Van Duyne R. P. (2009a). *Ad-Hoc* surface-enhanced raman spectroscopy methodologies for the detection of artist dyestuffs: thin layer chromatography-surface enhanced raman spectroscopy and *in situ* on the fiber analysis. Anal. Chem. 81, 3056–3062. 10.1021/ac802761v 19317457

[B2] Brosseau C. L. Rayner K. S. Casadio F. Grzywacz C. M. Van Duyne R. P. (2009b). Surface-enhanced raman spectroscopy: a direct method to identify colorants in various artist media. Anal. Chem. 81, 7443–7447. 10.1021/ac901219m 19637904

[B3] Bruni S. Guglielmi V. Pozzi F. (2010). Surface-Enhanced Raman Spectroscopy (SERS) on silver colloids for the identification of ancient textile dyes: tyrian purple and madder. J. Raman Spectrosc. 41, 175–180. 10.1002/jrs.2456

[B4] Bruni S. Guglielmi V. Pozzi F. Mercuri A. M. (2011). Surface-enhanced Raman Spectroscopy (SERS) on Silver colloids for the identification of ancient textile dyes. part ii: pomegranate and sumac. J. Raman Spectrosc. 42, 465–473. 10.1002/jrs.2736

[B5] Burnstock A. Lanfear I. van den Berg K. J. Carlyle L. Hendriks E. Kirby J. (2005). “A Comparison of the Fading and Surface Deterioration of Red Lake Pigments in Six Paintings by Vincent van Gogh with Artificially Aged Paint Reconstructions,” in 14th Triennial Meeting, The Hague, 12-16 September 2005: Preprints (ICOM Committee for Conservation) Triennial Meeting of the ICOM Committee for Conservation. London: James and James.

[B6] Cañamares M. V. Chenal C. Birke R. L. Lombardi J. R. (2008). DFT, SERS, and single-molecule SERS of crystal violet. J. Phys. Chem. C 112, 20295–20300. 10.1021/jp807807j

[B7] Cañamares M. V. Garcia-Ramos J. V. Domingo C. Sanchez-Cortes S. (2006). Surface-enhanced raman scattering study of the anthraquinone red pigment carminic acid. Vibr. Spectrosc. 40, 161–167. 10.1016/j.vibspec.2005.08.002

[B8] Cañamares M. V. Reagan D. A. Lombardi J. R. Leona M. (2014). TLC-SERS of mauve, the first synthetic dye. J. Raman Spectrosc. 45, 1147–1152. 10.1002/jrs.4508

[B9] Casadio F. Daher C. Bellot-Gurlet L. (2016). Raman spectroscopy of cultural heritage materials: overview of applications and new frontiers in instrumentation, sampling modalities, and data processing. Top. Curr. Chem. 374:62. 10.1007/s41061-016-0061-z 27573504

[B10] Casadio F. Leona M. Lombardi J. R. Van Duyne R. P. (2010a). Identification of organic colorants in fibers, paints, and glazes by surface enhanced raman spectroscopy. Acc. Chem. Res. 43, 782–791. 10.1021/ar100019q 20420359

[B11] Casadio F. Mauck K. Chefitz M. Freeman R. (2010b). Direct identification of early synthetic dyes: FT-raman study of the illustrated broadside prints of José Gaudalupe Posada (1852–1913). Appl. Phys. 100, 885–899. 10.1007/s00339-010-5668-2

[B12] Centeno S. A. Hale C. Carò F. Cesaratto A. Shibayama N. Delaney J. (2017). Van Gogh's *irises* and *roses*: the contribution of chemical analyses and imaging to the assessment of color changes in the red lake pigments. Her. Sci. 5:18 10.1186/s40494-017-0131-8

[B13] Cesaratto A. Leona M. Lombardi J. R. Comelli D. Nevin A. Londero P. (2014). Detection of organic colorants in historical painting layers using UV laser ablation surface-enhanced raman microspectroscopy. Angew. Chem. Int. Ed. 53, 14373–14377. 10.1002/anie.201408016 25353694

[B14] Cesaratto A. Lombardi J. R. Leona M. (2017). Tracking photo-degradation of triarylmethane dyes with surface-enhanced raman spectroscopy. J. Raman Spectrosc. 48, 418–424. 10.1002/jrs.5056

[B15] Cesaratto A. Londero P. Shibayama N. Lombardi J. R. Leona M. (2016). Fourier filtering ultraviolet laser ablation SERS for the analysis of yellow lakes. Microchem. J. 126, 237–242. 10.1016/j.microc.2015.12.018

[B16] Chen K. Leona M. Vo-Dinh K.-C. Yan F. Wabuyele M. B. Vo-Dinh T. (2006). Application of Surface-Enhanced Raman Scattering (SERS) for the identification of anthraquinone dyes used in works of art. J. Raman Spectrosc. 37, 520–527. 10.1002/jrs.1426

[B17] Corredor C. Teslova T. Cañamares M. V. Chen Z. Zhang J. Lombardi J. R. (2009). Raman and Surface-Enhanced Raman spectra of chrysin, apigenin and luteolin. Vibr. Spectrosc. 49, 190–195. 10.1016/j.vibspec.2008.07.012

[B18] Eastaugh N. Walsh V. Chaplin T. Siddall R. (2004). Pigment Compendium. A Dictionary of Historical Pigments. Oxford: Elsevier Butterworth-Heinemann.

[B19] Fang C. Agarwal A. Buddharaju K. D. Khalid N. M. Salim S. M. Widjaja E.. (2008). DNA detection using nanostructured SERS substrates with rhodamine B as raman label. Biosens. Bioelectron. 24, 216–221. 10.1016/j.bios.2008.03.032 18485693

[B20] Geiman I. Leona M. Lombardi J. R. (2009). Application of raman spectroscopy and surface-enhanced raman scattering to the analysis of synthetic dyes found in ballpoint pen inks. J. Forensic Sci. 54, 947–952. 10.1111/j.1556-4029.2009.01058.x 19457151

[B21] Greeneltch N. G. Davis A. S. Valley N. A. Casadio F. Schatz G. C. Van Duyne R. P.. (2012). Near-Infrared Surface-Enhanced Raman Spectroscopy (NIR-SERS) for the identification of eosin Y: theoretical calculations and evaluation of two different nanoplasmonic substrates. J. Phys. Chem. A 116, 11863–11869. 10.1021/jp3081035 23102210

[B22] Idone A. Gulmini M. Henry A.-I. Casadio F. Chang L. Appolonia L.. (2013). Silver colloidal pastes for dye analysis of reference and historical textile fibers using direct, extractionless, non-hydrolysis surface-enhanced raman spectroscopy. Analyst 138, 5895–5903. 10.1039/c3an00788j 23905159

[B23] Jeanmaire D. L. Van Duyne R. P. (1977). Surface raman spectroelectrochemistry: part I. heterocyclic, aromatic, and aliphatic amines adsorbed on the anodized silver electrode. J. Electroanal. Chem. Interf. Electrochem. 84, 1–20. 10.1016/S0022-0728(77)80224-6

[B24] Jensen L. Schatz G. C. (2006). Resonance raman scattering of rhodamine 6G as calculated using time-dependent density functional theory. J. Phys. Chem. A 110, 5973–5977. 10.1021/jp0610867 16671663

[B25] Jurasekova Z. Domingo C. Garcia-Ramos J. V. Sanchez-Cortes S. (2008). *In situ* detection of flavonoids in weld-dyed wool and silk textiles by surface-enhanced raman scattering. J. Raman Spectrosc. 39, 1309–1312. 10.1002/jrs.2053

[B26] Kirby J. White R. (1996). The Identification of Red Lake Pigment Dyestuffs and a Discussion of Their Use. In National Gallery Technical Bulletin, ed. Diana Davies and Jan Green, 17: 56–80. London: National Gallery Publications Limited.

[B27] Kneipp K. Wang Y. Kneipp H. Perelman L. T. Itzkan I. Dasari R. R. (1997). Single molecule detection using Surface-Enhanced Raman Scattering (SERS). Phys. Rev. Lett. 78, 1667–1670. 10.1103/PhysRevLett.78.1667

[B28] Leona M. (2005). “Sub-Nanogram Level Identification of Alizarin by Surface-Enhanced Raman Scattering,” in Proceedings Volume of the Sixth Infrared and Raman Users Group Conference, ed. M. Picollo, Florence: Il Prato.

[B29] Leona M. (2009). Microanalysis of organic pigments and glazes in polychrome works of art by surface-enhanced resonance raman scattering. Proc. Natl. Acad. Sci. U.S.A. 106, 14757–14762. 10.1073/pnas.0906995106 19667181 PMC2736431

[B30] Leona M. Lombardi J. R. (2007). Identification of berberine in ancient and historical textiles by surface-enhanced raman scattering. J. Raman Spectrosc. 38, 853–858. 10.1002/jrs.1726

[B31] Leona M. Stenger J. Ferloni E. (2006). Application of Surface-Enhanced Raman scattering techniques to the ultrasensitive identification of natural dyes in works of art. J. Raman Spectrosc. 37, 981–992. 10.1002/jrs.1582

[B32] Lin S. Hasi W.-L.-J. Lin X. Han S. Lou X.-T. Yang F. (2015). Rapid and sensitive SERS Method for determination of rhodamine B in chili powder with paper-based substrates. Anal. Methods 7, 5289–5294. 10.1039/C5AY00028A

[B33] Mayhew H. E. Fabian D. M. Svoboda S. A. Wustholz K. L. (2013). Surface-Enhanced raman spectroscopy studies of yellow organic dyestuffs and lake pigments in oil paint. Analyst 138, 4493–4499. 10.1039/c3an00611e 23722232

[B34] Murcia-Mascarós S. Domingo C. Sanchez-Cortes S. Cañamares M. V. Garcia-Ramos J. V. (2005). Spectroscopic identification of alizarin in a mixture of organic red dyes by incorporation in Zr-Ormosil. J. Raman Spectrosc. 36, 420–426. 10.1002/jrs.1315

[B35] Narayanan V. A. Stokes D. L. Vo-Dinh T. (1994). Vibrational spectral analysis of eosin Y and erythrosin B-intensity studies for quantitative detection of the dyes. J. Raman Spectrosc. 25, 415–422. 10.1002/jrs.1250250607

[B36] Narayanan V. A. Stokes D. L. Vo-Dinh T. (1996). Vibrational spectra of the industrial dyes cresyl fast violet, phloxine B and saffron. Intensity study by surface-enhanced raman spectroscopy. Analusis 24, 1–5.

[B37] Nie S. Emory S. R. (1997). Probing single molecules and single nanoparticles by surface-enhanced raman scattering. Science 275, 1102–6. 10.1126/science.275.5303.1102 9027306

[B38] Oakley L. H. Fabian D. M. Mayhew H. E. Svoboda S. A. Wustholz K. L. (2012). Pretreatment strategies for SERS analysis of indigo and prussian blue in aged painted surfaces. Anal. Chem. 84, 8006–8012. 10.1021/ac301814e 22897697

[B39] Pozzi F. Jan van den Berg K. Fiedler I. Casadio F. (2014). A systematic analysis of red lake pigments in french impressionist and post-impressionist paintings by Surface-Enhanced Raman Spectroscopy (SERS). J. Raman Spectrosc. 45, 1119–1126. 10.1002/jrs.4483

[B40] Pozzi F. Leona M. (2015). Surface-enhanced raman spectroscopy in art and archaeology. J. Raman Spectrosc. 47, 67–77. 10.1002/jrs.4827

[B41] Pozzi F. Lombardi J. R. Bruni S. Leona M. (2012b). Sample treatment considerations in the analysis of organic colorants by surface-enhanced raman scattering. Anal. Chem. 84, 3751–3757. 10.1021/ac300380c. 22462391

[B42] Pozzi F. Poldi G. Bruni S. De Luca E. Guglielmi V. (2012a). Multi-Technique characterization of dyes in ancient kaitag textiles from caucasus. Anthropol. Sci. 4, 185–197. 10.1007/s12520-012-0092-5

[B43] Pozzi F. Porcinai S. Lombardi J. R. Leona M. (2013a). Statistical methods and library search approaches for fast and reliable identification of dyes using Surface-Enhanced Raman Spectroscopy (SERS). Anal. Methods 5, 4205–12. 10.1039/c3ay40673c

[B44] Pozzi F. Shibayama N. Leona M. Lombardi J. R. (2013b). TLC-SERS study of syrian rue (*Peganum Harmala*) and its main alkaloid constituents. J. Raman Spectrosc. 44, 102–107. 10.1002/jrs.4140

[B45] Pozzi F. Zaleski S. Casadio F. Leona M. Lombardi J. R. Van Duyne R. P. (2016a). “Surface-Enhanced Raman Spectroscopy: Using Nanoparticles to Detect Trace Amounts of Colorants in Works of Art,” in Nanoscience and Cultural Heritage, eds. Ph. Dillmann, L. Bellot-Gurlet, and I. Nenner (Atlantis Press) 161–204. 10.2991/978-94-6239-198-7_6

[B46] Pozzi F. Zaleski S. Casadio F. Van Duyne R. P. (2016b). SERS discrimination of closely related molecules: a systematic study of natural red dyes in binary mixtures. J. Phys. Chem. C. 120, 21017–21026. 10.1021/acs.jpcc.6b03317

[B47] Roh J. Y. Matecki M. K. Svoboda S. A. Wustholz K. L. (2016). Identifying pigment mixtures in art using SERS: a treatment flowchart approach. Anal. Chem. 88, 2028–2032. 10.1021/acs.analchem.6b00044 26799174

[B48] Snowden M. J. Shadi I. T. Chowdhry B. Z. Snowden M. J. Withnall R. (2004). Semi-quantitative analysis of alizarin and purpurin by Surface-Enhanced Resonance Raman Spectroscopy (SERRS) using silver colloids. J. Raman Spectrosc. 35, 800–807. 10.1002/jrs.1199

[B49] Whitney A. V. Casadio F. Van Duyne R. P. (2007). Identification and characterization of artists' red dyes and their mixtures by Surface-Enhanced Raman Spectroscopy. Appl. Spectrosc. 61, 994–1000. 10.1366/000370207781745838 17910797

[B50] Whitney A. V. Van Duyne R. P. Casadio F. (2006). An innovative Surface-Enhanced Raman Spectroscopy (SERS) method for the identification of six historical red lakes and dyestuffs. J. Raman Spectrosc. 37, 993–1002. 10.1002/jrs.1576

[B51] Woodhead A. L. Cosgrove B. Church J. S. (2016). The purple coloration of four late 19th century silk dresses: a spectroscopic investigation. Spectrochim. Acta Part A 154, 185–192. 10.1016/j.saa.2015.10.024 26523685

[B52] Zaffino C. Bedini G. D. Mazzola G. Guglielmi V. Bruni S. (2016). Online coupling of high-performance liquid chromatography with surface-enhanced raman spectroscopy for the identification of historical dyes. J. Raman Spectrosc. 47, 607–615. 10.1002/jrs.4867

